# Multisensory stimuli elicit altered oscillatory brain responses at gamma frequencies in patients with schizophrenia

**DOI:** 10.3389/fnhum.2014.00788

**Published:** 2014-11-04

**Authors:** David B. Stone, Brian A. Coffman, Juan R. Bustillo, Cheryl J. Aine, Julia M. Stephen

**Affiliations:** ^1^The Mind Research Network and Lovelace Biomedical and Environmental Research InstituteAlbuquerque, NM, USA; ^2^Department of Psychology, Clinical Neuroscience Center, University of New MexicoAlbuquerque NM, USA; ^3^Department of Psychiatry, Health Sciences Center, University of New MexicoAlbuquerque, NM, USA; ^4^Department of Radiology, Health Sciences Center, University of New MexicoAlbuquerque, NM, USA

**Keywords:** schizophrenia, multisensory, audio-visual, gamma, time-frequency, event-related oscillations, MEG, magnetoencephalography

## Abstract

Deficits in auditory and visual unisensory responses are well documented in patients with schizophrenia; however, potential abnormalities elicited from multisensory audio-visual stimuli are less understood. Further, schizophrenia patients have shown abnormal patterns in task-related and task-independent oscillatory brain activity, particularly in the gamma frequency band. We examined oscillatory responses to basic unisensory and multisensory stimuli in schizophrenia patients (*N* = 46) and healthy controls (*N* = 57) using magnetoencephalography (MEG). Time-frequency decomposition was performed to determine regions of significant changes in gamma band power by group in response to unisensory and multisensory stimuli relative to baseline levels. Results showed significant behavioral differences between groups in response to unisensory and multisensory stimuli. In addition, time-frequency analysis revealed significant decreases and increases in gamma-band power in schizophrenia patients relative to healthy controls, which emerged both early and late over both sensory and frontal regions in response to unisensory and multisensory stimuli. Unisensory gamma-band power predicted multisensory gamma-band power differently by group. Furthermore, gamma-band power in these regions predicted performance in select measures of the Measurement and Treatment Research to Improve Cognition in Schizophrenia (MATRICS) test battery differently by group. These results reveal a unique pattern of task-related gamma-band power in schizophrenia patients relative to controls that may indicate reduced inhibition in combination with impaired oscillatory mechanisms in patients with schizophrenia.

## Introduction

On a moment-by-moment basis, behaviorally salient information reaches us from multiple senses and seamlessly shapes our perceptions and actions in the world. The capacity to organize the many stimuli arriving from different sensory modalities into a coherent and operable percept remains one of the marvels of the central nervous system. Despite a rich history exploring unisensory processes in the brain, understanding the processes involved in multisensory integration remains a central challenge in systems neuroscience.

Empirical and computational approaches have revealed some of the neural mechanisms subserving multisensory integration at the cellular and systems level. Based on both human and animal studies, it is clear that a broad network of cortical (sensory and association areas—Barth et al., 1995; Schroeder et al., 2001; Falchier et al., 2002; Brett-Green et al., 2003) and subcortical (superior colliculus—Meredith and Stein, 1986; Wallace et al., 1993, 1998; Peck, 1996; Bell et al., 2001) areas are involved in multisensory processing (Ghazanfar and Schroeder, 2006). Both non-invasive functional neuroimaging and invasive studies have confirmed either facilitation or suppression of unisensory responses with multisensory stimuli both at the single cell (Meredith and Stein, 1986; Kadunce et al., 1997; Fu et al., 2003) and at the population level (Barth et al., 1995; Molholm et al., 2002, 2006; Murray et al., 2005; Stone et al., 2011) indicating that multisensory responses often do not represent a simple linear summation of the unisensory responses. In conjunction with these neurophysiological results, behavioral studies also confirm that multisensory stimuli lead to facilitation of unisensory reaction times and improvements in accuracy under certain conditions (Calvert et al., 2004). Additional studies provide evidence that multisensory integration is dependent upon both bottom-up sensory features, such as salience of sensory stimuli (Stein and Meredith, 1993), and top-down cognitive processes, including attention (Busse et al., 2005; Talsma et al., 2007; Keitel et al., 2013).

However, the mechanism by which sensory information from independent sensory modalities is integrated to generate the unified percept is still poorly understood. Certain brain regions contain cells that react to input from multiple sensory modalities (e.g., superior colliculus, association areas). Though studies examining these multisensory cells have played an important role in advancing research in this area, research at the cellular level does not provide a complete view of multisensory integration. Gamma-band oscillations (>30 Hz), in particular, are implicated in aspects of feature binding (e.g., linking objects within a visual scene) by establishing temporal synchrony both within and across cortical regions (Tallon-Baudry et al., 1996; Von Stein and Sarnthein, 2000). For example, conscious perception of stimuli is accompanied by increases in oscillatory synchrony at frequencies above 30 Hz (Melloni et al., 2007). Additional results provide evidence that changes in gamma-band power are associated with perceptual and cognitive processing (Bertrand and Tallon-Baudry, 2000; Tallon-Baudry, 2009). Multisensory studies have further established that gamma-band oscillations play a role in cross-modal feature binding in both animal and human studies (Senkowski et al., 2007; Ghazanfar et al., 2008; Chandrasekaran and Ghazanfar, 2009; Chandrasekaran et al., 2011). For example, Ghazanfar et al. (2008) identified increased gamma-band coherence between auditory cortex and the superior temporal sulcus in response to multisensory relative to unisensory stimuli in Rhesus monkeys. Furthermore, using local field potential recordings in the superior temporal sulcus of macaques, Chandrasekaran and Ghazanfar (2009) determined that gamma oscillations demonstrated robust multisensory facilitation whereas multisensory facilitation in other frequency bands was dependent on the timing between auditory and visual stimuli. Finally, both humans and non-human primates demonstrate the same multisensory behavioral facilitation in parallel tasks (Chandrasekaran et al., 2011) implying similar mechanisms are employed across species. The effort to better understand the role of oscillations in multisensory processing in the human brain has been expanded further by the application of functional neuroimaging techniques (for a review, see Stein and Stanford, 2008). Both Kaiser et al. (2005) and Yuval-Greenberg and Deouell (2007) identified greater gamma-band power in response to congruent vs. incongruent multisensory stimuli. Furthermore, Mishra et al. (2007) determined that perception of the AV flash illusion (Shams et al., 2002) was accompanied by bursts of gamma oscillations.

Schizophrenia is accompanied by both sensory and cognitive deficits in conjunction with the core symptoms associated with the disorder (Adcock et al., 2009; Javitt, 2009). Recent studies have indicated that cognitive factors are more directly related to quality of life than symptom alleviation (Green et al., 2004). Correlations between sensory deficits and cognitive functioning are found in multiple studies (Uhlhaas et al., 2008; Bedwell et al., 2011; Silverstein and Keane, 2011) and impaired processing of sensory information likely contributes to cognitive dysfunction in schizophrenia. Multisensory integration provides a bridge between unisensory and cognitive processing by requiring activation of a broader cortical network without requiring explicit cognitive skills. Our previous study (Stone et al., 2011) indicates that multisensory stimuli may benefit patients with schizophrenia relative to healthy controls with both behavioral and neurophysiological multisensory facilitation observed in patients with schizophrenia in a forced choice paradigm despite unisensory deficits in patients with schizophrenia. Although neurophysiological and behavioral multisensory facilitation were both observed in patients, these effects were not directly correlated. In contrast, Williams et al. (2010) determined that schizophrenia patients had less behavioral facilitation than controls using a multisensory detection task. These results differ from our previous study employing a forced choice paradigm. Furthermore, De Gelder et al. (2002) reported no difference in multisensory reaction time between schizophrenia patients and controls, providing consistency with our results. In sum, results are currently mixed in terms of how patients with schizophrenia process multisensory stimuli relative to controls with few multisensory neuroimaging studies focused on this population.

Changes in gamma-band power have been observed in schizophrenia both at rest and during task execution (for recent reviews, see Gandal et al., 2012; Uhlhaas and Singer, 2012). Many of the gamma-band differences reported in schizophrenia relative to controls have been elicited in response to auditory steady state stimuli showing reduced gamma-band activity and reduced hemispheric asymmetry (Kwon et al., 1999; Hamm et al., 2011; Tsuchimoto et al., 2011). Because perceptual and cognitive deficits are core impairments in schizophrenia, individuals suffering from the disorder may be especially prone to disruptions in multisensory processing (De Jong et al., 2010; Williams et al., 2010; Stone et al., 2011). Evidence suggests that reductions in auditory induced gamma-band power may reflect impaired sensory responsiveness due to increased resting-state gamma-band activity (Teale et al., 2008; Wilson et al., 2008; Spencer, 2011); however, the relationship between multisensory processing and gamma-band oscillations in schizophrenia has yet to be reported. The goal of the current study is to investigate the link between event-related gamma-band oscillations and multisensory integration in patients with schizophrenia and healthy controls.

To address this question, we recruited schizophrenia patients (SP) and healthy controls (HC) to perform a multisensory integration task while brain activity was recorded using magnetoencephalography (MEG). A simple multisensory paradigm was employed by presenting both unisensory (auditory-A and visual-V) stimuli as well as multisensory (AV) stimuli within a forced choice reaction time task. Based on the previously reported deficits in gamma-band oscillations, we hypothesized that gamma-band power would be reduced in SP relative to HC. The task also required that the participants identify the location of the stimulus within a perspective drawing, thereby presenting participants with near and far stimuli which corresponded to both peripherally- and centrally-presented visual stimuli, respectively. Peripherally-presented visual stimuli preferentially activate the dorsal visual stream (Ungerleider and Desimone, 1986; Livingstone and Hubel, 1987; Stephen et al., 2002) which is impaired in schizophrenia (Butler and Javitt, 2005; Koychev et al., 2011). Therefore, we hypothesized that patients would show greater deficits in gamma-band power in response to peripheral (dorsal stream) visual stimuli relative to central (ventral stream) visual stimuli. Furthermore, multisensory studies have demonstrated that patients with schizophrenia have a wider window of integration than healthy controls when the stimuli are offset in time (Foucher et al., 2007), suggesting that HC can differentiate asynchronous stimuli with smaller delays between auditory and visual stimuli than SP. In summary, we hypothesized that SP and HC would show differential patterns of event-related gamma-band activity and these differences would vary by condition (unisensory/multisensory, near/far, and synchronous/asynchronous).

## Materials and methods

### Participants

Participants in the study included 103 individuals (46 SP and 57 age-matched HC). All participants provided written informed consent prior to study procedures. All procedures were performed in accordance with the Declaration of Helsinki and with prior approval from the University of New Mexico Health Sciences Center Human Research Review Committee. All SP met DSM-IV criteria for a diagnosis of schizophrenia or schizoaffective disorder, were stable on their medications for at least 1 month prior to study participation, and were periodically assessed throughout study enrollment to confirm clinical and pharmacological stability. HC and their first-degree relatives possessed no prior history of any psychiatric disorder based on the SCID-NP. None of the participants suffered from substance abuse, prior head trauma, or other neurological disorders, based on a standard neurological exam. Participant demographics are provided in Table 1. As part of recruitment into the study, participants' neurocognitive abilities were assessed using the MATRICS test battery. This battery is designed to measure neurocognitive impairments, which present as core deficits in schizophrenia (Kern et al., 2008; Nuechterlein et al., 2008).

**Table 1:** **Participant demographics**.

	**HC**	**SP**
Gender	40 males/17 females	39 males/7 females
Age	39.4 (12.7) years	39.2 (13.9) years
IQ	111.9^*^ (11.3)	101.5^**^ (17.1)
Medication	-	14.5^**^ (7.5) mg/day
Positive symptoms	-	15.5 (4.9)
Negative symptoms	-	14.6 (5.2)

*IQ based on WASI Neuropsychological Test; Medication is based on olanzapine equivalent dosage; Positive and Negative symptoms are cumulative scores from PANSS symptom scale; Values in parentheses represent standard deviations*.

* *Mean based on 56 controls*.

** *Means based on 44 patients*.

### Behavioral task

**Figure 1:**
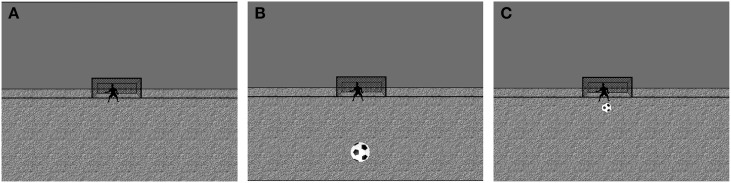
**Task stimuli**. The background presented in **(A)** was present throughout the entire task performance. The participants were instructed to fixate on the goalie during the task. Auditory stimuli were presented with the visual background in place. During trials with visual stimuli the only change to the visual scene was the addition of a soccer ball in one of two locations Near **(B)** or Far **(C)**. During multisensory conditions the visual stimulus was presented in combination with the auditory tone.

To assess responses to unisensory and multisensory stimuli, participants performed a simple stimulus discrimination task. During the task, participants were presented with ecologically relevant audio-visual stimuli designed to mimic the image of a soccer ball and the sound of a soccer ball “bounce.” As control conditions, visual-only (the soccer ball image) and auditory-only (the soccer ball bounce sound) stimuli were also presented. During the presentation of all stimuli, participants viewed a static background on a projection screen positioned at a distance of 1 m from nasion. A simplified soccer field with a goalie and net provided a perspective-drawing framework, and participants were asked to fixate upon the goalie during all stimulus presentations (Figure 1A). There were two visual-only, two auditory-only, and four audio-visual stimulus presentations yielding eight distinct stimulus conditions:

*The visual-only near stimulus condition (VN):* During the VN condition, the image of a black and white soccer ball appeared on the static soccer field background for 200 ms. The ball was centered at 8° below fixation and subtended 2.7° of visual angle, giving the appearance that the ball was “downfield” and near to the participant (Figure 1B). This condition activates peripheral visual field associated with dorsal stream processing.

*The visual-only far stimulus condition (VF):* During the VF condition, the soccer ball image appeared on the soccer field at 1.8° below fixation and subtended 1° of visual angle, giving the impression that the ball was “upfield” and farther away from the participant. The ball remained on the screen for 200 ms (Figure 1C). The VF and VN stimuli were scaled according to Rovamo and Virsu (1979) to account for the cortical magnification factor. This condition activates central visual field associated with ventral stream processing.

*The auditory-only near stimulus condition (AN):* During the AN condition, a 550 Hz tone was presented binaurally for 200 ms through a set of ear plugs at a volume of 63 dB above hearing threshold. Threshold was determined uniquely for each participant prior to task performance. No other visual stimuli were presented during the AN condition, other than the static soccer field background (Figure 1A).

*The auditory-only far stimulus condition (AF):* The AF condition was identical to the AN condition except that the tone was presented at a lower volume of 45 dB above hearing threshold in order to mimic a more distant sound.

*The audio-visual near stimulus condition (AVSN):* The AVSN condition consisted of the presentation of the VN stimulus followed by the AN stimulus with a 5 ms delay.

*The audio-visual far stimulus condition (AVSF):* During the AVSF condition, the VF stimulus was presented and followed by the AF stimulus after a 5 ms delay.

*The audio-visual asynchronous near stimulus condition (AVAN) and The audio-visual asynchronous far stimulus condition (AVAF):* The final two audio-visual conditions were identical to the AVN and AVF conditions except that the delay between the visual and auditory stimuli was increased from 5 to 50 ms to emulate the natural delay between the two stimulus types which occur at a distance from the participant due to the differential speed of light and sound.

During each trial, one of the eight stimulus conditions was randomly presented. Participants were asked to indicate whether the stimulus was near to them or farther away by pressing one of two buttons on a response device with either their right index finger or right middle finger, respectively. During 20% of the trials, feedback regarding response accuracy was given. If the response was correct on an audio-visual trial, the image of the ball rolled into the soccer goal along with the sound of a cheering crowd. If an incorrect response or no response was given the ball rolled away from the goal accompanied by a “groaning crowd” sound. Unisensory trial feedback was matched to the sensory modality (i.e., only the rolling ball for visual conditions, or the crowd reaction for auditory conditions). Trial duration was between 1500 and 1900 ms and varied randomly from trial to trial, rendering an inter-stimulus interval (ISI) between 1300 and 1700 ms. Trials were organized into six blocks of 200 trials, separated by short breaks, where each condition was randomly presented with equal probability such that there were approximately 150 trials of each condition. Participants received pre-recorded instructions and were given a brief practice run to ensure that they understood the task prior to MEG data collection. The entire task, including auditory threshold determination, instructions, the practice run and the six trial blocks, was performed in a magnetically shielded room while participants were seated in the MEG chair. MEG and behavioral data used in the analyses were collected during the six trial blocks.

### MEG data collection and processing

MEG data were collected with a whole head, 306-channel Elekta Neuromag system located at the Mind Research Network in Albuquerque, NM and data were acquired at a sampling rate of 1000 Hz with a 0.1 Hz high-pass and 330 Hz low-pass anti-aliasing filter. To permit comparisons between participants and groups, MEG sensor data for each participant were interpolated to the same reference head position using Neuromag Maxfilter software (Taulu et al., 2004; Taulu and Kajola, 2005) during post-processing. The reference head position was chosen based on the average head location across participants within the study. Maxfilter software also eliminates noise from sources that originate from outside of the defined head volume including muscle artifact and non-physiological flux jumps identified by an automated algorithm (Taulu et al., 2004; Taulu and Simola, 2006). Eyeblinks were eliminated from the data using a projector based on the average eye blink for each subject (Uusitalo and Ilmoniemi, 1997). MEG data for each participant were epoched for each condition over an interval from 500 ms preceding stimulus onset until 500 ms after onset. Trials with incorrect responses and trials where the magnetic field exceeded 7 pT in any MEG gradiometer were also rejected. SP had significantly more trials rejected for incorrect responses, so HC trials were culled by randomly removing trials until both groups had an equal number of trials for each condition. Results are based upon an average of 142 ± 10 trials/condition for HC and 144 ± 8 trials/condition for SP. Preprocessing was performed using the scriptable MNE preprocessing pipeline (martinos.org/mne).

### Event-related oscillation analysis

Linear trends, which spanned the 1000 ms time-window, were removed prior to time-frequency transformation. Each 1000 ms trial was then converted to the time-frequency domain using Morlet wavelets applied to each MEG gradiometer (width = 7 cycles, frequency range = 7–50 Hz). Baseline-corrected spectral maps were computed by frequency for each trial in decibels (dB), with average spectral power from −100 to 0 ms relative to stimulus onset as the measure of baseline noise. Spectral power at each MEG gradiometer, time point, and frequency was then averaged across trials for each condition and participant. Based on the spatial specificity of planar gradiometers (Ahonen et al., 1993), we only analyzed the planar gradiometer data (magnetometer data is not reported here). To facilitate processing and reduce the number of comparisons, spectral power from the two planar gradiometers that occupied the same MEG sensor location was summed for all sensor pairs using the Fieldtrip function “ft_combineplanar.” Thus, there were 102 combined gradiometers (henceforth referred to simply as sensors) used for analysis. All time-frequency analyses were performed using Fieldtrip (Oostenveld et al., 2011) and custom MATLAB programs (MathWorks, Inc., Natick, MA, USA).

### Statistical analysis

#### Behavioral data

To assess significant differences in accuracy and reaction time (RT), three-way multivariate analysis-of-variance tests (MANOVAs) were performed on the reaction times and accuracy (% correct) for the unisensory and multisensory stimuli. In these tests, stimulus type (auditory-only, visual-only, or audio-visual) and stimulus location (near or far) were treated as within-subject factors, while group identification (SP or HC) was treated as a between-subjects factor. Reaction times were only assessed for correct responses. Separate three-way MANOVAs were performed comparing reaction times and accuracy in which synchrony (synchronous or asynchronous) and stimulus location were treated as within-subjects factors and group identification was treated as a between-subjects factor. When significant main or interaction effects were detected, Bonferroni-corrected *t*-tests were performed to more closely examine the nature of these effects.

#### Event-related oscillation data

Event-related oscillations were determined by identifying significant increases or decreases in baseline-corrected spectral power within subject group in the 0–480 ms time window. Significance was determined by one-sample *t*-tests using FDR correction with *q* = 0.05. Group differences in spectral power were confirmed to overlap with the time/frequency windows of significant increases or decreases in power relative to baseline in at least one group, and those which did not overlap were excluded from further analysis. No group differences were found which did not overlap with event-related oscillations showing either significant increases or decreases in power relative to baseline.

Spectral power was compared between HC and SP across the 30–50 Hz (gamma-band) frequency range from 0 to 480 ms post-stimulus for each condition. We limited our time window to 0–480 ms to focus on event-related oscillations following stimulus presentation. We focused our analysis on the 30–50 Hz gamma-band range based on unisensory and multisensory studies reporting results in this frequency range. Independent sample *t*-tests comparing SP to HC were applied at each sensor, time, and frequency point. The results of these *t*-tests were then used to identify candidate clusters of significant group differences in gamma-band power. Candidate clusters were identified when at least 3 adjacent channels, 3 adjacent frequency points, and 20 adjacent time points were significantly different at the *p* < 0.05 threshold. This criterion alone limits spurious results as described by Guthrie and Buchwald (1991) when data are highly correlated. Following the recommendation of Maris and Oostenveld (2007), further testing to limit Type I errors associated with multiple comparisons was performed through permutation testing (repeated analyses with random shuffling of SP and HC) of each candidate sensor-time-frequency cluster to determine if the candidate group differences were statistically unlikely to have been observed by chance (*p* < 0.05). For these tests, participants' data were randomly re-assigned group identification (shuffled) while keeping the number of SP and HC constant. *T*-tests were applied to the data points in the identified clusters using these new group identifications. Group permutations were performed 5253 times (the number of unique permutations of 103 binary numbers) for each cluster and *t*-statistics were calculated and summed for each cluster for each permutation. When this summed *t*-statistic after permutation exceeded the summed *t*-statistic from the candidate cluster in more than 5% of the permutations, the cluster was rejected as not significantly different by group and was excluded from further analysis.

#### Regression and correlation analyses

Stepwise multiple regressions were performed separately for HC and SP to assess the extent to which mean spectral power of unisensory clusters predicted mean spectral power of multisensory clusters while limiting comparisons to the same stimulus location (near or far). Regression analyses were only performed for the clusters, which differed significantly from baseline values within group. Additionally, regressions were performed by group to determine if gamma-band power in each of the clusters predicted multisensory reaction time facilitation and MATRICS composite *t*-scores for subtests of interest (processing speed, attention, verbal learning, visual learning, and working memory). Regression models were considered significant at *p* < 0.025 (0.05 divided by the number of multisensory clusters) to correct for multiple comparisons.

Finally, Pearson correlation was used to assess the relationships among mean spectral power of unisensory clusters, medication dosage (in Olanzapine equivalent dose), and positive/negative symptoms (as assessed by the Positive and Negative Symptoms [PANS] scale).

## Results

### Behavioral results

#### Reaction times

**Figure 2:**
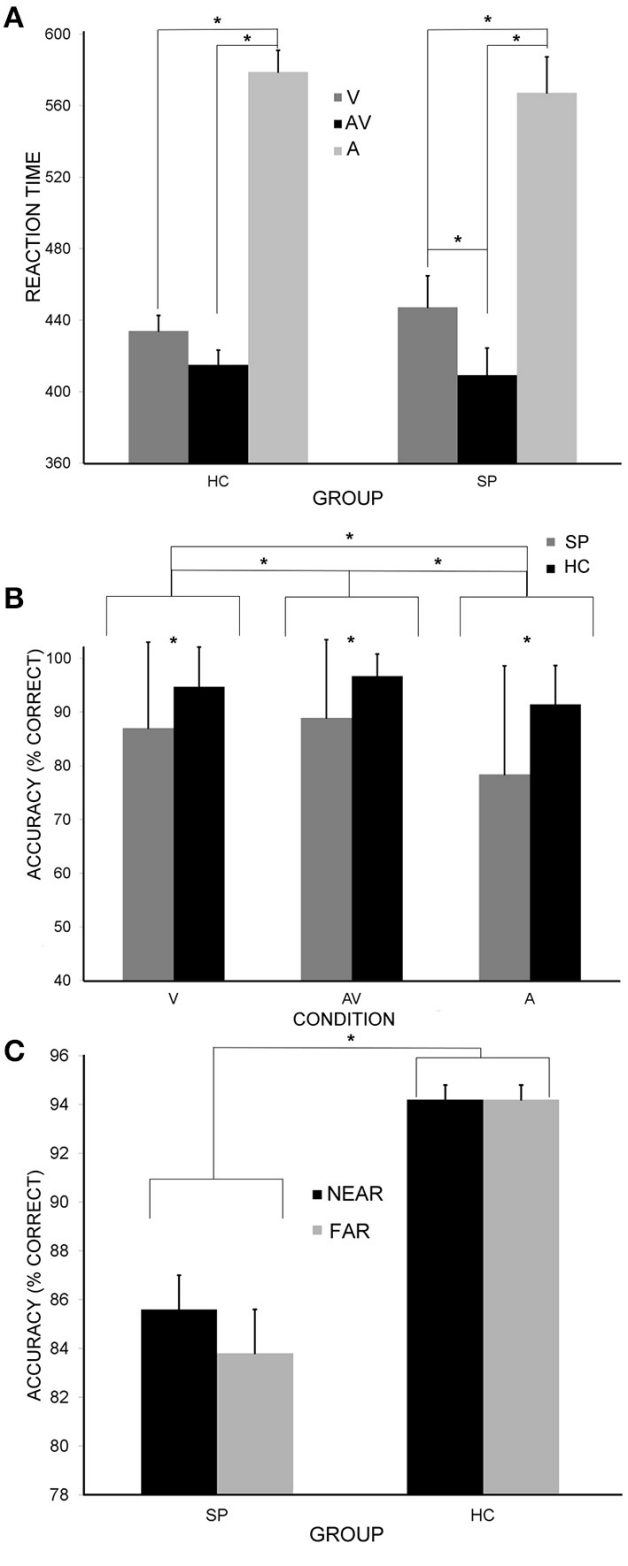
**Behavioral differences. (A)** Mean reaction times for the SP and HC group to unisensory (A and V) and multisensory (AV) stimuli. Significant main effects and interactions are denoted by asterisks. In addition to a main effect of condition, there was a group by condition interaction showing that SP had greater multisensory facilitation than HC—the fastest unisensory response (V) was significantly slower in SP, yet the AV RTs were equivalent by group. **(B)** Accuracy (% correct responses) for each group in response to unisensory (A and V) and multisensory (AV) stimuli. HC had significantly more correct responses to all three stimulus types compared to SP. **(C)** Accuracy for each group in response to near and far stimuli collapsed across conditions. HC had significantly more correct responses to both near and far stimuli compared to SP. SP had a significantly greater difference in near and far response accuracy compared to HC. Error bars represent s.e.m.

Table 2 and Figure 2 display group mean reaction times and accuracy results for each condition. A main effect of stimulus type was detected [*F*_(2, 99)_ = 410.26, *p* < 0.001] in which reaction times to visual-only and audio-visual stimuli were significantly faster when compared to auditory-only stimuli [*t*_(101)_ = 17.06; *t*_(101)_ = 24.27, respectively; *p* < 0.001, both tests]. Audio-visual stimuli also evoked faster reaction times than visual-only stimuli [*t*_(101)_ = 9.06; *p* < 0.001]. By comparing the AV RTs to the fastest unisensory RTs (V for both HC and SP), this result indicates significant multisensory facilitation based on average RT. A significant stimulus type by group interaction was also detected [*F*_(2, 99)_ = 5.25; *p* = 0.007], in which differences between visual-only and audio-visual responses were significantly greater for SP than HC [*t*_(100)_ = 3.24; *p* = 0.002; Figure 2A]. This result provides evidence of differential multisensory facilitation by group with SP showing greater improvement in AV RTs relative to V RTs.

**Table 2:** **Behavioral means**.

	**HC**	SP^*^	**HC**	SP^*^
	**Reaction time**		**Accuracy (% Correct)**	
VN	428.6 (9.0)	439.7 (17.6)	95.1 (0.7)	88.4 (1.5)
VF	439.3 (8.6)	454.9 (17.8)	94.2 (0.8)	85.5 (1.8)
AN	579.3 (12.7)	565.1 (21.0)	90.4 (0.8)	78.0 (1.9)
AF	578.7 (11.0)	569.7 (19.6)	92.4 (0.7)	78.8 (2.4)
AVSN	406.8 (8.6)	399.2 (14.8)	97.2 (0.4)	90.5 (1.2)
AVSF	423.1 (8.4)	419.6 (15.4)	96.2 (0.5)	87.2 (1.8)
AVAN	425.2 (9.1)	415.0 (15.0)	97.2 (0.4)	91.5 (1.2)
AVAF	442.2 (8.6)	437.9 (16.1)	95.8 (0.5)	87.7 (1.6)

*Means (s.e.m.) of reaction times and accuracy*.

* *Behavioral data missing for one patient. Excluded from table and all behavioral analyses*.

A significant effect of stimulus location (near vs. far) was also detected [*F*_(1, 100)_ = 19.51, *p* < 0.001], where RTs to near stimuli were significantly faster. Additionally, there was a significant stimulus type by stimulus location interaction [*F*_(2, 99)_ = 8.25; *p* < 0.001], in which RTs to the VN condition were significantly faster than RTs to the VF condition [*t*_(101)_ = 4.28; *p* < 0.001]. Likewise AVN RTs were faster than AVF RTs [*t*_(101)_ = 6.48; *p* < 0.001].

A significant main effect of audio-visual synchrony was detected when comparing AVN/AVF RTs to AVAN/AVAF RTs. Responses to synchronous presentations were faster than asynchronous presentations [*F*_(1, 100)_ = 160.75; *p* < 0.001]. No significant group effects were detected based on the synchrony of the AV stimuli (all *p*'s > 0.05).

#### Response accuracy

The accuracy comparisons yielded similar results. There was a significant main effect of stimulus type [*F*_(2, 99)_ = 122.42; *p* < 0.001], where more correct responses occurred in the visual-only and audio-visual conditions compared to the auditory-only condition [*t*_(101)_ = 7.50; *t*_(101)_ = 13.37, respectively; *p* < 0.001, both cases]. There were also more correct responses to audio-visual presentations when directly compared to visual-only presentations [*t*_(101)_ = 3.96; *p* < 0.001]. A significant stimulus type by group interaction was found [*F*_(2, 99)_ = 12.17; *p* < 0.001] such that HC had significantly more correct responses to all stimulus types compared to SP [visual-only *t*_(101)_ = 29.62; auditory-only *t*_(101)_ = 28.78; audio-visual *t*_(101)_ = 41.15; *p* < 0.001, all cases; Figure 2B]. This interaction provides evidence that both groups showed improved accuracy for the AV condition relative to the unisensory conditions.

There was also a significant main effect of stimulus location [*F*_(1, 100)_ = 4.87; *p* = 0.03], where more correct responses were made for near stimuli. A significant stimulus location by group interaction was also detected [*F*_(2, 99)_ = 4.93; *p* = 0.03], in which HC had significantly more correct responses to both near stimuli [*t*_(100)_ = 6.20; *p* < 0.001] and far stimuli [*t*_(100)_ = 5.81; *p* < 0.001; Figure 2C]. Finally, a significant stimulus type by stimulus location interaction was detected [*F*_(2, 99)_ = 14.49; *p* < 0.001], where more correct responses were found for VN than VF stimuli and AVN than AVF stimuli [*t*_(101)_ = 4.48; *t*_(101)_ = 4.58, respectively; *p* < 0.001, both cases]. This provides evidence that the peripheral visual stimuli and higher volume auditory stimuli lead to improved accuracy in this task and the combined AV condition improved accuracy, especially for the near condition.

### Event-related gamma-band oscillation results

**Figure 3:**
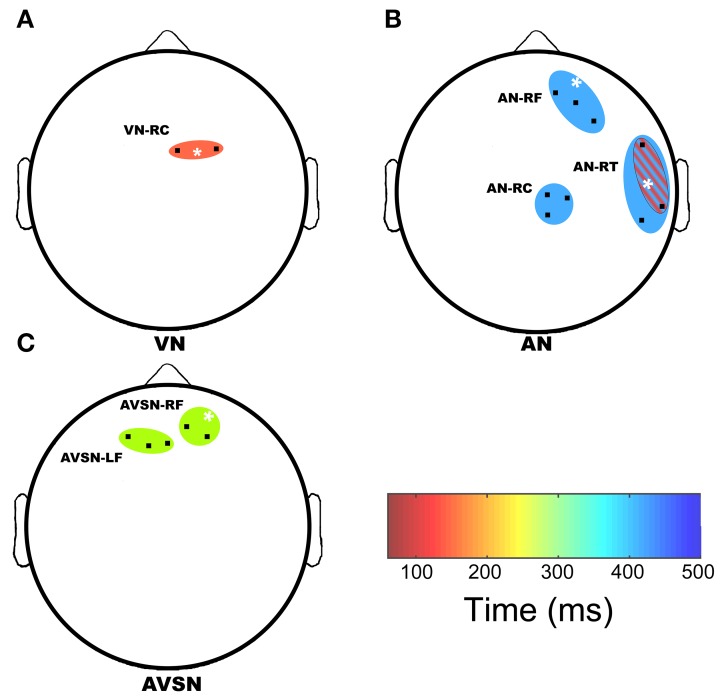
**Regions of significant group differences**. A top down view of the sensor array is presented to show regions of significant group differences in gamma-band power. The top of the plot denotes frontal regions whereas left and right correspond to left and right temporal regions with occipital regions located at the bottom of the plot. The colored ovals denote where group differences in gamma-band power relative to baseline were detected in the VN **(A)**, AN **(B)**, and AVSN **(C)** conditions. Black points within each colored oval mark sensor locations where time-frequency differences emerged. The ovals are color-coded to represent time (in ms) when significant differences occurred in each region. Two regions of significant group differences overlapped spatially; AN-RT1 is shown with red diagonal stripes overlaid on AN-RT2. The white asterisk denotes the time frequency plot that is displayed in Figure 4. LF, left frontal; RF, right frontal; RC, right central; RT, right temporal.

**Figure 4:**
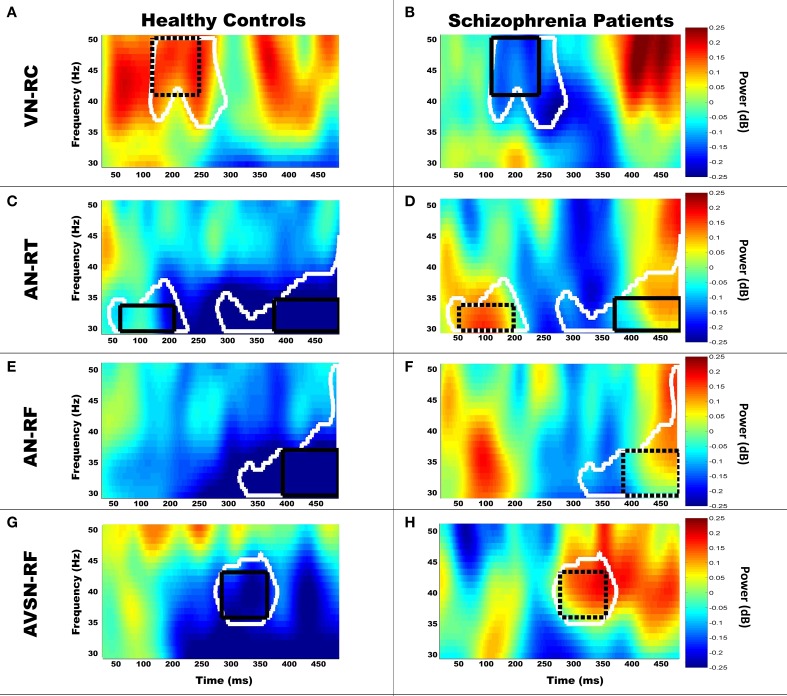
**Example time-frequency maps for each condition**. Group-averaged baseline-corrected time-frequency power maps (scale in dB) for a representative sensor of 5/7 clusters with significant group differences (Table 3) are displayed. Group comparisons are displayed by comparing the left (HC) and right (SP) columns. The black boxes denote the overlapping region of significant group differences for the location presented in Figure 3 across the three regional channels. Dotted boxes denote regions which did not show significant within-group differences from baseline, while solid boxes denote those which do show significant within-group differences relative to baseline. The white outline denotes the region that showed significant group differences within the displayed channel. As expected the overlap across regional channels is smaller than the significant region for the displayed channel due to spatial variation of oscillatory activity. Three of the clusters of significant group differences indicate increased gamma-band power in SP. AN-RT1 (early) and AN-RT2 (late) are both displayed in the AN-RT plots. Group-averaged time frequency plots of gamma band power are displayed for region VN-RC **(A,B)**, AN-RT **(C,D)**, AN-RF **(E,F)**, and AVSN-RF **(G,H)**.

**Figure 5:**
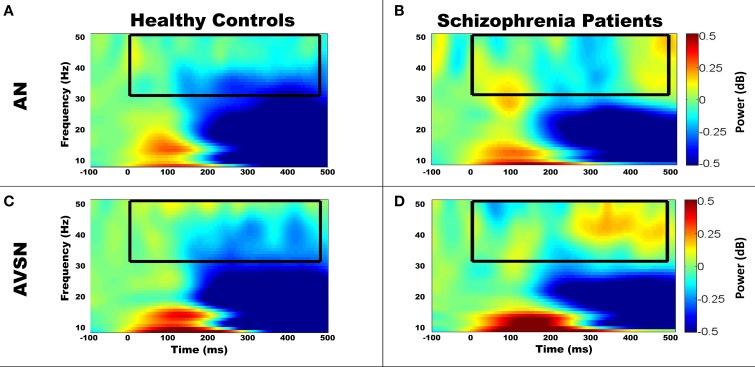
**Example time frequency maps showing task-related responses**. The group averaged time frequency maps showed the expected increases in low frequency power demonstrating a clear task-related response to stimuli over the relevant brain regions. To display the broader task-related response we display the time frequency map from 7 to 50 Hz and from −100 to 500 ms. For example, right temporal locations showed a response to the AN stimulus **(A,B)**—see zoomed in time frequency plot in Figures 4C,D. Furthermore, AVSN-RF time frequency plots (**C,D**—correspond to Figures 4G,H) located over right frontal region are shown. The black box denotes the time-frequency window that was analyzed in this study and displayed in Figure 4. Note the change in scale between this figure and Figure 4 due to the larger changes in power generally observed at lower frequencies. Clear changes from baseline are observed by 100 ms poststimulus in response to the auditory/visual stimuli below 20 Hz.

Permutation testing of the candidate channel-time-frequency clusters revealed regions of significant group differences in gamma-band power, which are summarized in Table 3 and Figures 3 and 4. Figure 3 depicts regions and time intervals where group differences in gamma-band power were found while Figure 4 shows the time-frequency power maps for the representative sensor where group differences were identified (denoted by the white asterisk in Figure 3). Significant group differences were found only in near unisensory and synchronous multisensory conditions. All clusters demonstrating significant group differences were confirmed to show significant increases or decreases in gamma-band power relative to the prestimulus time period in the FDR-corrected one-sample *t*-test comparisons within one or both diagnostic groups. This provides evidence of task-related increases or decreases in gamma-band power in either one or both groups in each of the group difference clusters (see asterisks in Table 3). Examples of the full time-frequency maps (7–50 Hz, −100–500 ms) are presented in Figure 5 with the analysis region (30–50 Hz, 0–480 ms) for group comparisons highlighted with a black box. A clear event-related response is present in both the alpha- (~10 Hz) and gamma-band (30–50 Hz) frequency ranges.

**Table 3:** **Clusters of significant group differences in gamma-band Power**.

**Cluster label^†^**	**MEG channel**	**Latency (ms)**	**Frequency (Hz)**	**Group difference**	**Cluster *t*-value**	**Cluster *p*-value**
VN-RC	1041	96–280	37–50	SP^*^ < HC	−2.71	0.019
	1111	98–252	36–50			
	1121	0–247	33–50			
AN-RT1	1441	0–274	30–36	SP > HC^*^	2.61	0.020
	2611	15–174	30–37			
	2621	38–147	30–38			
AN-RT2	1441	349–480	30–36	SP^*^ > HC^*^	3.03	0.032
	2611	233–480	30–45			
	2621	244–480	30–50			
	2641	349–480	30–37			
AN-RC	0731	248–480	30–37	SP > HC^*^	2.51	0.024
	2211	338–480	30–50			
	2241	321–480	30–37			
AN-RF	0921	279–480	30–50	SP > HC^*^	2.98	0.022
	0931	312–480	30–41			
	0941	367–480	30–39			
	1231	367–480	30–37			
AVSN-LF	0541	232–362	34–50	SP^*^ > HC^*^	2.60	0.031
	0611	199–480	34–50			
	1011	222–480	30–46			
AVSN-RF	0921	229–355	35–46	SP > HC^*^	2.41	0.031
	0931	242–334	36–43			
	0941	242–391	30–46			

† *Clusters are listed with the stimulus condition, followed by the sensor region where they were detected. LF, left frontal; RF, right frontal; RC, right central; RT, right temporal*;

* *indicates gamma power that significantly deviated from baseline using the 1-sample FDR-corrected test*.

There were five clusters with significant group differences in gamma-band power in response to unisensory stimuli (see Figures 3A,B, 4A–F), which were located over frontal, central and temporal brain regions. The time windows of these group differences in gamma-band power ranged from early (e.g., AN-RT1) to late (e.g., AN-RC) and spanned the 30–50 Hz range (see Table 3).

During multisensory stimulus presentation there were two clusters showing significant group differences in gamma-band power located over frontal cortex (Figures 3C, 4G,H). The timing of these differences in gamma-band power was restricted to the latter half of the analysis window (>225 ms poststimulus), and the location, time window and frequency range of one of these clusters (AVSN-RF) overlapped with unisensory differences in the auditory cluster AN-RF.

### Unisensory and multisensory cluster regressions

Table 4 displays the significant results from multisensory cluster regressions for HC based on clusters that deviated significantly from baseline values. There was a clear difference in predictive power between SP and HC. In HC, gamma-band power in multisensory clusters was predicted by unisensory clusters restricted to the right central sensors, whereas none of the gamma band clusters that deviated significantly from zero for SP predicted multisensory gamma. Finally, gamma-band power did not predict RT facilitation for HC or SP.

**Table 4:** **Significant multisensory cluster regressions for HC**.

**Regressands**	**Regressors**	**Beta (β)**	**Partial correlation**	***R*^2^**	***p***
AVSN-LF	AN-RC	0.28	0.43	0.26	< 0.001^*^
AVSN-RF	AN-RC	0.30	0.35	0.12	0.008^*^
RT-fac. (AVSN)	None				

* *These p-values were significant with correction for multiple comparisons (α = 0.05/2 = 0.025). RT-fac, Reaction Time multisensory facilitation relative to fastest unisensory RT*.

### Neurocognitive regressions

Table 5 displays group means and standard errors for the MATRICS subtests. As expected, SP performed significantly worse on all measures relative to HC.

**Table 5:** **MATRICS scores**.

	**HC**		**SP**	
	**Mean (s.e.m.)**	n^*^	**Mean (s.e.m.)**	n^*^
Processing speed^**^	53.5 (1.2)	55	36.9 (1.9)	45
Attention/Vigilance^**^	49.0 (1.3)	51	37.6 (2.1)	45
Working memory^**^	50.1 (1.3)	55	42.2 (1.9)	45
Verbal learning^**^	45.9 (1.2)	55	39.0 (1.3)	45
Visual learning^**^	45.7 (1.4)	55	38.0 (1.7)	45

*SEM, Standard Error of the Mean*.

* *Not all participants completed each MATRICS domain and their data for that domain were excluded from the table and regression analyses*.

** *Significant at p < 0.001*.

Tables 6 and 7 display results of neurocognitive regressions for HC and SP, respectively. For HC, working memory was predicted by VN-RC, and verbal learning was predicted by AVSN-LF gamma band power, both with negative relationships. For SP, VN-RC predicted attention, working memory, and verbal learning, all with negative relationships. VN-RC was always the first to enter the model when it was predictive of MATRICS scores; however, AN-RT2 predicted working memory when accounting for variance explained by VN-RC. Within SP, medication dosage was positively correlated with AVSN-LF (*r* = 0.33, *p* = 0.031).

**Table 6:** **Significant neurocognitive regressions for HC**.

**Regressands**	**Regressors**	**Beta (β)**	**Partial correlation**	***R*^2^**	***p***
MATRICS Processing speed	None				
MATRICS Attention	None				
MATRICS Working memory	VN-RC	−7.1	−0.27	0.71	0.049
MATRICS Verbal learning	AVSN-LF	−9.9	−0.27	0.74	0.044
MATRICS Visual learning	None				

**Table 7:** **Significant neurocognitive regressions for SP**.

**Regressands**	**Regressors**	**Beta (β)**	**Partial correlation**	***R*^2^**	***p***
MATRICS Processing speed	None				
MATRICS Attention	VN-RC	−17.8	−0.39	0.26	0.002
	AN-RT2	10.3	0.37		
MATRICS Working memory	VN-RC	−16.2	−0.38	0.20	0.010
MATRICS Verbal learning	VN-RC	−9.4	−0.32	0.10	0.032
MATRICS Visual learning	None				

## Discussion

The aim of this study was to determine if differences in 30–50 Hz gamma-band power in response to unisensory and multisensory stimuli in a large cohort of schizophrenia patients relative to age-matched healthy controls explained the multisensory facilitation observed in our previous study. Similar to our previous study, this larger cohort of SP showed greater behavioral facilitation to multisensory stimuli than HC. However, gamma-band power was not directly associated with RT facilitation. Despite this lack of association with RT, gamma-band power predicted performance on MATRICS scores differently by group suggesting that gamma-band power may play a role in cognitive deficits in SP. Also, medication was positively correlated with gamma-band power for only one of the multisensory clusters suggesting that medication alone cannot account for the differences in gamma-band power. Furthermore, SP showed decreases in gamma-band power in the peripheral visual (VN) condition relative to HC, as hypothesized. In contrast to our hypothesis, we identified both *decreases* and *increases* in gamma-band power in SP relative to HC. These group differences in multisensory gamma-band power were not directly accounted for by group differences in unisensory gamma-band power (e.g., the unisensory and multisensory group differences in gamma-band power did not simultaneously overlap in time, frequency, and location). Despite the lack of spatio-temporal overlap, certain unisensory clusters predicted gamma-band power in multisensory clusters indicating a cortical network of local gamma-band power that may influence gamma-band power in other regions. Finally, synchrony of the AV stimuli modulated group differences such that group differences were only obtained for the synchronous multisensory conditions. These results are discussed in more detail below.

Our results are consistent with the previous literature (Senkowski et al., 2008) suggesting that gamma-band oscillations play a role in multisensory integration by identifying task-related increases and decreases in gamma-band power. Interestingly, despite the greater behavioral improvement in SP relative to HC (more facilitation of multisensory RTs relative to unisensory RTs), gamma-band power did not predict multisensory RT facilitation for either HC or SP. Previous studies indicated that increased gamma was associated with conscious recognition of the multisensory flash illusion (Mishra et al., 2007) and stimuli with greater salience (e.g., looming AV stimuli—Maier et al., 2008); whereas other studies only found multisensory effects in other frequency bands (e.g., theta-band Naue et al., 2011). Based on our current results, gamma-band oscillations do not play a direct role in facilitating multisensory RTs in SP. However, there is considerable variability in RTs in both HC and SP and single-trial analysis (not feasible in low SNR non-invasive studies) may be required to confidently conclude that gamma oscillations do not play a role in behavioral RT measures. Finally, Xu et al. (2013) determined that oscillatory activity across multiple frequency bands including gamma (30–50 Hz) provided excellent (91% accuracy) discrimination between SP and HC groups during lexical processing. This may indicate that despite a lack of behavioral correlates, group differences in gamma-band oscillations may provide a means to better differentiate groups.

Perhaps the most surprising result from the current study is that 6 out of 7 clusters showed greater gamma-band power in SP relative to HC. However, in a number of cases (4/6) this represented a failure to suppress gamma-band power following the stimulus in SP rather than a significant increase from baseline gamma-band power. Only 2 of the clusters (AN-RT2 and AVSN-LF) represented increased gamma-band power from baseline in SP as well as significantly greater gamma-band power than HC. Increased gamma-band power in the AVSN-LF cluster may in part be explained by the positive correlation between gamma-band power and medication level in SP. Significant differences in gamma-band power in schizophrenia have been observed in a number of paradigms, but reports of decreases in gamma-band power in SP are more common than increases (for a review, see Sun et al., 2011). It is important to note that some of these studies limited their analysis to specific regions of interest (e.g., Teale et al., 2008; Oribe et al., 2010), thereby limiting the scope of the study to the region analyzed. Our analysis limited the frequency range to the low gamma-band, yet performed comparisons across the full sensor array providing a broader view of changes in gamma-band power. Also, the regions that showed increased gamma in SP relative to HC (anterior temporal and frontal regions) are consistent with previous reports of increased gamma-band power in frontal regions in SP as summarized in a review by Sun et al. (2011). Furthermore, Tikka et al. (2013) reported increased gamma-band power (30–50 Hz) in unmedicated schizophrenia patients with minor physical anomalies relative to controls over right frontal, temporal and parietal regions, similar to our findings. The remaining clusters with group differences showing SP > HC were associated with significant decreases in gamma-band power in HC (AN-RT1, AN-RC, AN-RF, and AVSN-RF) in which each cluster included auditory stimuli. Haenschel et al. (2009) found both increases and decreases in frontal gamma activity in SP and HC, depending on working memory load. While our task was not specifically designed as a working memory task *per se*, it required that participants maintain a representation of “Near” and “Far” stimuli to perform the discrimination in the fully randomized design. In conjunction with Haenschel and colleagues' results, this may indicate that the current task is tantamount to a low-demand working memory task for healthy controls but requires additional effort for SP, which is accompanied by increased gamma in these patients. In support of this hypothesis, SP performed more poorly than HC for the auditory discrimination, which was more difficult than the visual discrimination, and gamma-band power increases in SP were observed during the auditory and multisensory task.

To further characterize the role of unisensory processing deficits on multisensory gamma responses, we also investigated whether unisensory gamma-band power was predictive of multisensory gamma-band power. The non-linear transformation of the Morlet wavelet eliminates the ability to directly compare gamma-band power between A+V vs. AV (Senkowski et al., 2007), as is commonly performed in multisensory evoked response studies (Calvert and Thesen, 2004). Therefore, we assessed the influence of unisensory gamma-band power on multisensory gamma-band power through regression analyses. Different patterns of unisensory gamma predicting multisensory gamma are demonstrated in Table 4 for HC (relative to no predictors in SP). AN-RC predicted gamma-band power for AVSN-RF and AVSN-LF. In both cases the prediction showed a positive relationship suggesting that increased unisensory gamma-band power predicted increases in multisensory gamma-band power. However, the absence of a relationship between gamma-band power and RT may be related to the variability in the gamma response relative to RT.

As hypothesized, group differences were noted based on the location of the visual stimulus in the visual field (central-Far vs. peripheral-Near). Our results only reveal group differences in gamma-band power during the peripheral—Near visual conditions. These results are consistent with previous studies indicating dorsal stream deficits in SP (Butler and Javitt, 2005; Butler et al., 2008). In this case SP showed significant decreases in gamma-band power relative to baseline and relative to HC; this result may indicate that gamma-band deficits in SP may contribute to impaired peripheral field processing in SP. Additionally, group differences were only found with synchronous presentation of auditory and visual stimuli. This result is contrary to our hypothesis that group differences would be obtained during the asynchronous condition due to differences in sensitivity to the temporal integration window (differences in the response to asynchrony of the AV stimuli—Foucher et al., 2007). This may indicate group differences in how gamma-band oscillations bind auditory and visual stimuli. Parametric manipulations of temporal synchrony are needed to better understand this result in relation to schizophrenia.

Finally, we investigated whether event-related power in the gamma-band clusters was predictive of cognitive outcome on five subtests of the MATRICS, and whether it was related to symptomology in SP. As shown in Tables 6 and 7, gamma-band power predicted performance on the MATRICS for both HC and SP. Surprisingly, in most cases the relationship indicated that increased gamma-band power negatively correlated with MATRICS scores (in all cases for HC). Furthermore, multisensory gamma-band power only showed a relationship with MATRICS scores (verbal learning) in HC. On the other hand, only unisensory gamma-band power predicted MATRICS scores in SP. Unlike HC, the SP group showed an association between gamma-band power and MATRICS attention scores, with both AN and VN gamma-band power predicting the attention score. Yet, visual gamma-band power negatively predicted attention scores, whereas auditory gamma-band power positively predicted gamma-band power when controlling for visual gamma-band power. These results suggest that alterations in unisensory processing impact cognitive abilities in SP, consistent with previous visual and auditory studies (Butler et al., 2008; Uhlhaas et al., 2008; Smith et al., 2010).

The underlying pathophysiology responsible for the gamma-band differences observed in this study and others could arise from a number of sources. GABAergic interneurons have been implicated in the generation and modulation of gamma-band oscillations in cortex and hippocampus (Traub et al., 1996; Whittington et al., 2000), and recent evidence suggests a critical role for GABA during audio-visual integration in rodents (Iurilli et al., 2012). Furthermore, GABA synthesis is reduced in individuals with schizophrenia, and reduced GABA transmission in prefrontal cortex has been associated with cognitive deficits including working memory deficits (Akbarian et al., 1995a,b). Yet another study found indications of decreased GABA_B_ in schizophrenia (Farzan et al., 2010), which plays a role in modulating gamma oscillations. This alteration in GABA_B_ may explain increases in gamma-band power in SP as an improper inhibition of cortical oscillations. Another potential source of gamma-band differences is reduced or altered connectivity within and between brain areas in schizophrenia. Disruptions in anatomical and functional brain networks have been observed in individuals with schizophrenia (Burns et al., 2003; Zhou et al., 2007), and direct connectivity between sensory areas and other cortical regions has been implicated in generating the oscillatory changes observed during cross-modal stimulus presentations (Lakatos et al., 2007). Based on the differences in gamma-band power in response to auditory and multisensory stimuli, this may indicate disconnection of auditory cortex in SP, thereby leading to altered local gamma-band power. Therefore, disordered cortical pathways may contribute to the group differences in gamma-band power observed in these results.

Our results extend previous work characterizing multisensory differences in task-related activity in schizophrenia (Stone et al., 2011), and add to the effort to better understand the causes and consequences of schizophrenia. However, we recognize certain limitations exist in the current study that bear consideration and highlight the direction of future research. In this initial characterization, we have limited our analysis to the 30–50 Hz gamma-band range; however, differences in other frequency bands likely exist. Evidence suggests that different frequencies are associated with different cognitive processes (for a review, see Ward, 2003). For example, high gamma (>60 Hz) has been implicated in higher cognitive processes (Uhlhaas et al., 2011). Exploring differences in other frequency bands and their interactions during multisensory processing in schizophrenia will be the focus of future work. Furthermore, unlike EEG, MEG does not suffer from propagation of artifacts from the reference electrode. Yet, it would be beneficial to extend the current results to a source-based analysis of oscillatory activity to better understand the network interplay associated with multisensory processing. Furthermore, directly linking these multisensory gamma differences to unisensory processing through empirical methods suggested by Senkowski et al. (2007) will allow us to better understand how differences in unisensory processing influence multisensory abilities in both HC and SP. Medication effects and differences in cognitive ability between groups further hamper our ability to fully understand the underlying aspects of schizophrenia independent of these confounds. A recent study by Tikka et al. (2013) indicated increased gamma-band power in unmedicated patients with schizophrenia relative to controls; however, medication levels only corresponded to gamma-band power in one of the group clusters, suggesting that this alone cannot explain the current results. Finally, Sivarao et al. (2013) reported that nicotine appears to enhance gamma-band power associated with the auditory steady-state response in rats. Nicotine is a known confound in the current study with decreased access to HC who smoke based on smoking cessation programs, thereby limiting the ability to match on this factor in large studies. As mentioned by Sivarao and others, nicotine may provide a means for SP to self-medicate and normalize brain function.

In summary, this study demonstrates that group differences between SP relative to HC in gamma-band activity are present in response to both unisensory and multisensory stimuli. Yet, the unisensory deficits do not directly map onto changes in gamma-band power in response to multisensory stimuli. Future work will tease apart the role of sensory parameters and attention in performing unisensory and multisensory tasks. Characterizing oscillations across the frequency spectrum will also likely provide broader insight into multisensory integration in schizophrenia and help provide a link between sensory and cognitive functions.

### Conflict of interest statement

The authors declare that the research was conducted in the absence of any commercial or financial relationships that could be construed as a potential conflict of interest.
